# Supplementary material for: DeepMP: a deep learning tool to detect DNA base modifications on Nanopore sequencing data
Source: Bioinformatics. 2021 Oct 28;38(5):1235–43. doi: 10.1093/bioinformatics/btab745 (PMC8826383; doi:10.1093/bioinformatics/btab745)
Supplement: btab745_Supplementary_Data [file btab745_supplementary_data.pdf]

Supplementary material for "DeepMP: a deep learning tool to detect DNA base modifications on Nanopore sequencing data"

Jose Bonet[1,2,†, *], Mandi Chen[4,5,†, *], Marc Dabad[6,7], Simon Heath[6,7], Abel Gonzalez-Perez[1,2], Nuria Lopez-Bigas[1,2,3], and Jens Lagergren[4,5]

[1]Institute for Research in Biomedicine (IRB Barcelona), The Barcelona Institute of Science and Technology, 08028 Barcelona, Spain
[2]Research Program on Biomedical Informatics, Universitat Pompeu Fabra, Barcelona, Catalonia, Spain
[3]Institució Catalana de Recerca i Estudis Avançats (ICREA), Barcelona, Spain
[4]Department of Electrical Engineering and Computer Science, KTH Royal Institute of Technology, Stockholm, Sweden
[5]Science for Life Laboratory, Stockholm, Sweden
[6]CNAG-CRG, Centre for Genomic Regulation (CRG), Barcelona Institute of Science and Technology (BIST), Barcelona, Spain
[7]Universitat Pompeu Fabra (UPF), Barcelona, Spain
[†]Both Authors contributed equally to this study
[*]Corresponding authors: jose.bonet@irbbarcelona.org | mandiche@kth.se

1 Supplementary Methods

1.1 Benchmarking methylation detection with similar tools

Similarly to [15], we benchmarked DeepMP against four existing tools: Nanopolish [13], Megalodon [10], DeepSignal [8], and Guppy [9].

Nanopolish (v0.13.2) [13] assigns a log-likelihood ratio to each CpG site or group of nearby CpG sites that share the same methylation level. A positive log-likelihood ratio value indicates evidence of methylation. Groups of CpGs were separated to include all the predictions per read and site. Additionally, a site was considered to be methylated if the log-likelihood was above 2.0 and unmethylated if it was lower than -2.0 [3]. Finally, log-likelihood values were transformed into probabilities for the benchmarking. Methylation calling through Nanopolish was performed following the steps presented by Simpson et al. in [4].

DeepSignal (v0.1.7) [8] is an RNN-based model to detect the methylation state of a given motif. It renders information about the methylated status of the motif per read and site. If the probability of methylation was above 0.5, the position in the read was considered to be methylated. Otherwise, called unmethylated. Models for DeepSignal were trained and tested in the same sets as DeepMP.

Guppy (v4.4.2) [9] directly basecalls 5mC at CpG sites as a fifth base along the four canonical DNA bases. The configuration file `dna_r9.4.1_450bps_modbases_dam-dcm-cpg_hac.cfg` was used as Guppy's modified basecalling model. The reads produced incorporate the information supporting the modifications at a given genomic position. We then used the scripts provided at [1] to convert the guppy methylation calls from fast5 files to reference anchored files. As in nanopolish, resulting log-likelihood values were transformed into probabilities for the benchmarking.

Megalodon (v2.3.2) [10] uses Guppy to re-basecall the fast5 reads to then identify methylated motifs by aligning the basecalling output to the reference. It assigns a score for the candidate modified base and performs a calibration when converting the raw scores to estimated empirical probabilities. We employed the most recent basecalling models in Rerio [11] to detect CpG (`res_dna_r941_min_modbases_5mC_CpG_v001.cfg`) as well as methylation in GATC motifs (`res_dna_r941_min_modbases-all-context_v001.cfg` with Megalodon. Megalodon produces per-read modified base log probability and canonical base log probability at each mapped site of the motif. For the benchmarking analysis per site and read, we transformed the resulting log-likelihoods into probabilities.

Additional tools were not included for the following reasons. We decided not to include DeepMod [6] because DeepSignal, with a model with similar architecture, has been proven to outperform it [15]. Similarly, SignalAlign [12] has shown lower performance than DeepSignal [8]. Besides, the model has not been updated in recent years. We did not consider mCaller [7], because only models trained for 6mA and not for 5mC were available. Finally, Tombo [14] was not included in the analysis as it is not considered the state-of-the-art method to detect methylations by their developers, Oxford Nanopore Technologies. Furthermore, recent studies have shown reduced performance when compared against the methods employed in the presented benchmarking [15]. On the other hand, Epinano [5], a method to detect modifications based on the basecalling error information, was discarded due to its inability to call methylated sites per read.

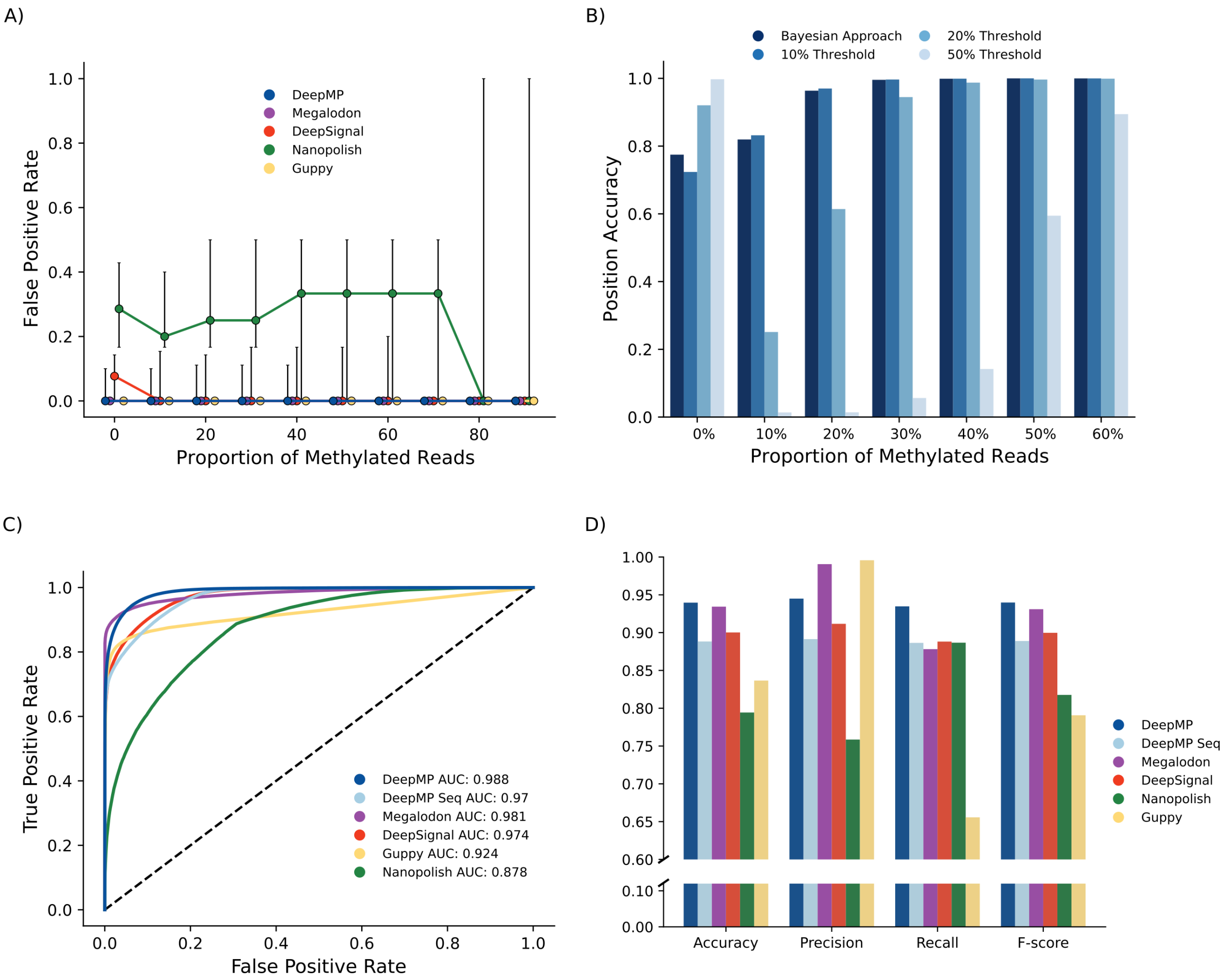

Figure S1: **A)** False positive rate (y-axis) evaluated on eleven datasets comprising different levels of methylation (0-100%) (x-axis) on DeepMP, Megalodon, DeepSignal, Guppy and Nanopolish. **B)** Comparison of the position accuracy of DeepMP at different thresholds and proportion of methylated reads against the position-based calling Bayesian approach. **C)** Receiver operating characteristic (ROC) curve showing the false positive rate (x-axis) vs. the true positive rate (y-axis) for the read predictions on a mixture of methylated and unmethylated reads in a single cross validation fold. **D)** Accuracy measurements for the models on the cross validation fold presented in C.

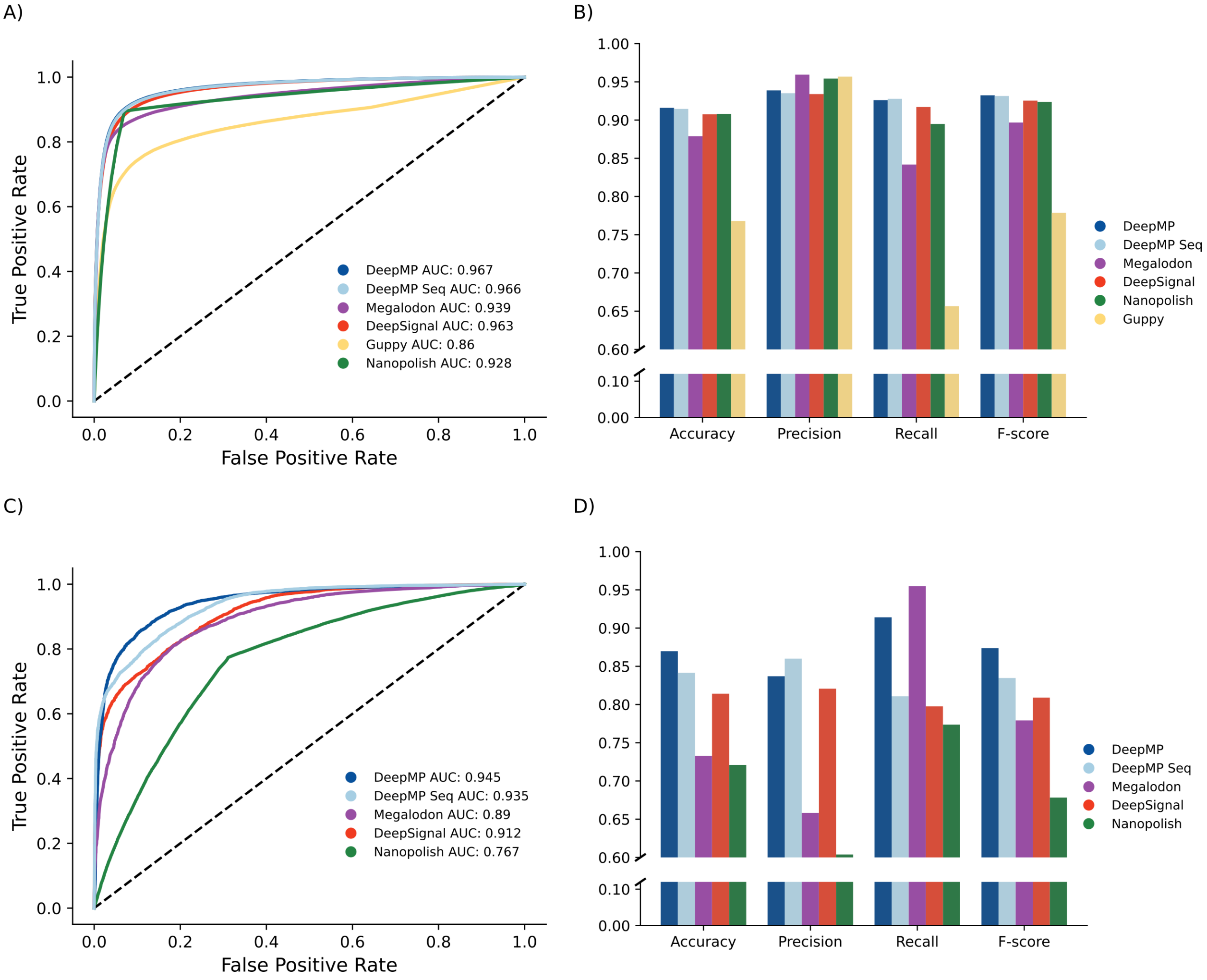

Figure S2: Performance of DeepMP, DeepMP Seq, Megalodon, Guppy, DeepSignal and Nanopolish on the Norwich subset of the human dataset and of DeepMP, DeepMP Seq, Megaldon, DeepSignal and Nanopolish on the pUC19 plasmid. **A, C)** Receiver operating characteristic (ROC) curve showing the false positive rate (x-axis) vs. the true positive rate (y-axis) for the read predictions on a mixture of methylated and unmethylated reads on the human dataset (A) and pUC19 (C) respectively. **B)** Accuracy measurements for the models on the human dataset. **D)** Accuracy measurements for the models on the pUC19 plasmid

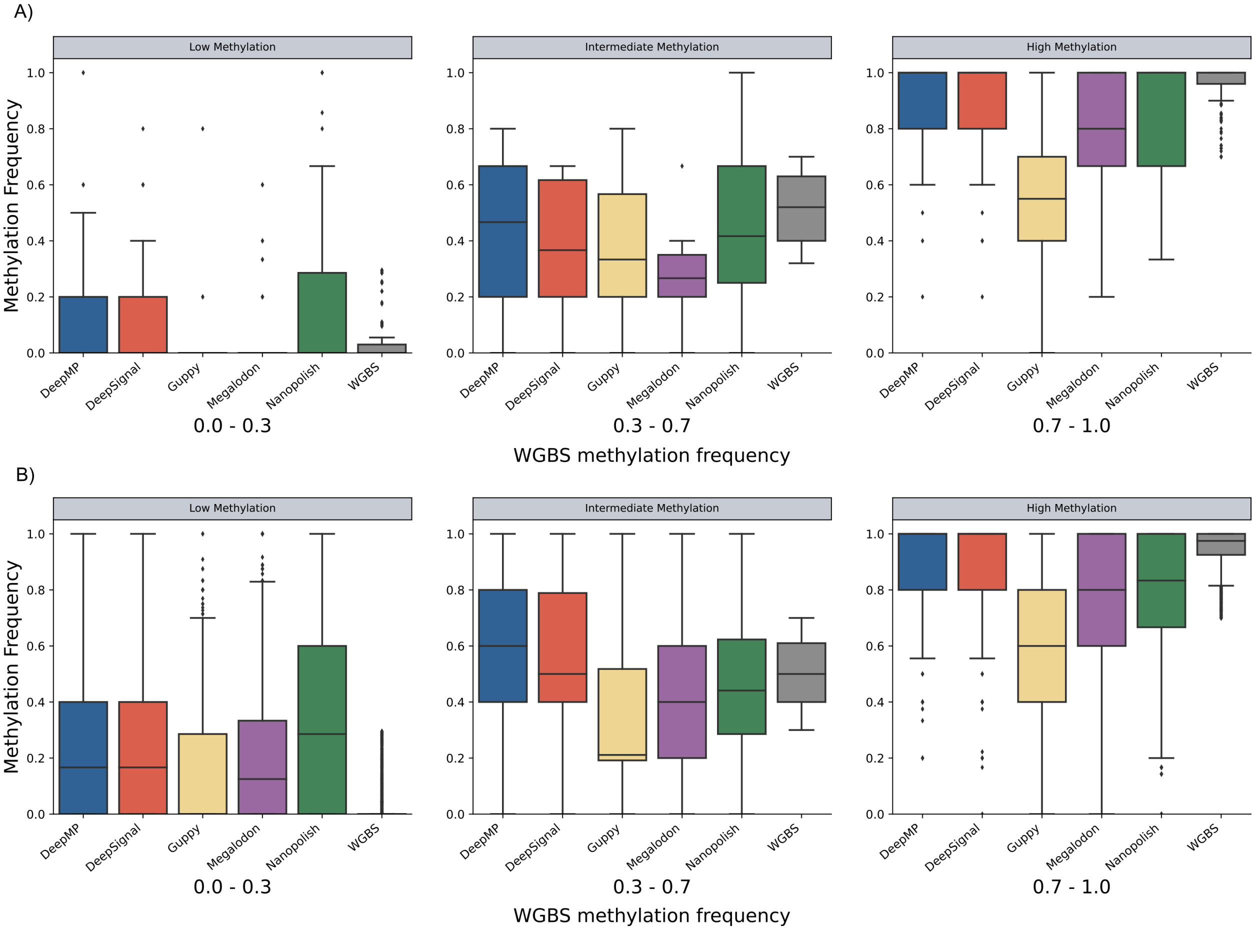

Figure S3: Distribution of methylation calls from nanopore (y axis) across three WGBS methylation bins: low methylation (0.0–0.3), intermediate methylation (0.3–0.7), and high methylation (0.7–1.0) for imprinting genes (**A**) and LINE1 regions (**B**) for DeepMP, DeepSignal, Guppy, Megalodon and Nanpolish.

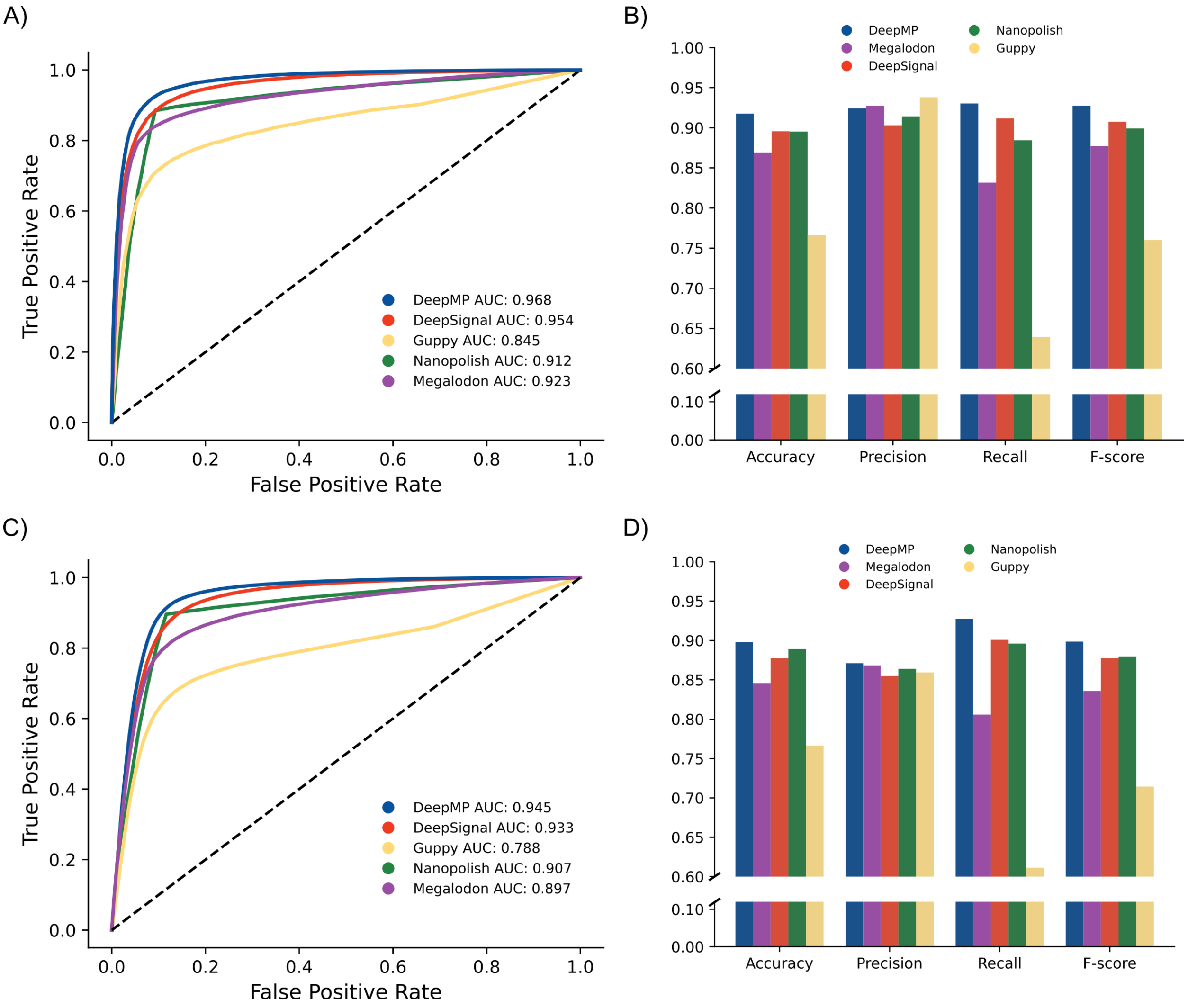

Figure S4: Performance of DeepMP, Megalodon, Guppy, DeepSignal and Nanopolish on the Norwich subset of the human dataset for Imprinting genes (A, B) and LINE1 regions (C, D). **A, C)** Receiver operating characteristic (ROC) curve showing the false positive rate (x-axis) vs. the true positive rate (y-axis) for the read predictions on a mixture of methylated and unmethylated reads on imprinting genes (A) and LINE1 regions (C) respectively. **B)** Accuracy measurements for the models on imprinting genes. **D)** Accuracy measurements for the models on LINE1 regions

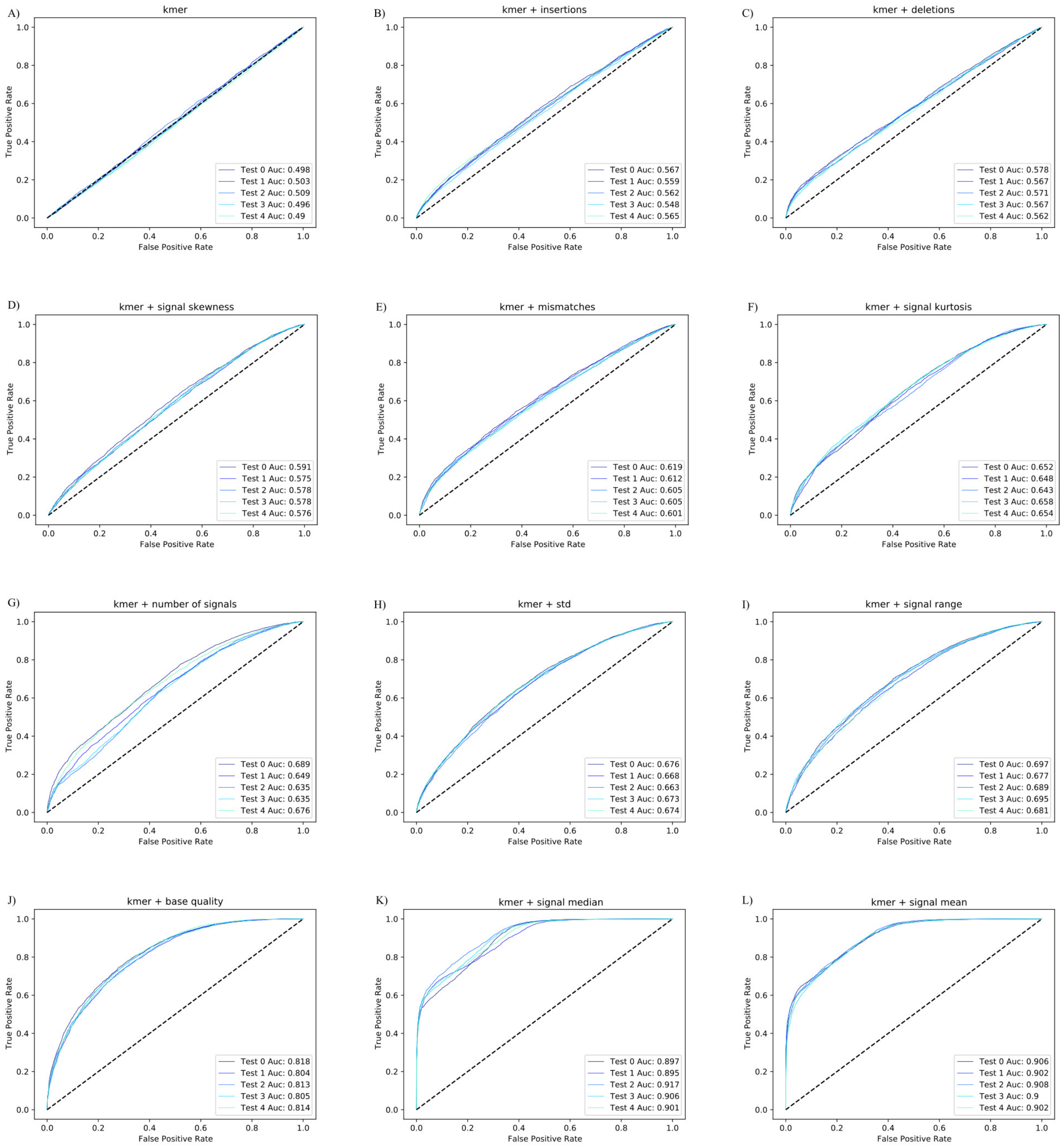

Figure S5: Single feature performance on a subset of the *E. coli* dataset. Kmer serves as a baseline feature and it is combined with all other individual features. Performance on **A)** kmer alone, **B)** insertions, **C)** deletions , **D)** signal skewness, **E)** mismatches, **F)** signal kurtosis, **G)** number of signals, **H)** signal std, **I)** signal range, **J)** base quality, **K)** signal median and **L)** signal mean. **A-L)** Receiver operating characteristic (ROC) curve showing the false positive rate (x-axis) vs. the true positive rate (y-axis) for the read predictions on a mixture of methylated and unmethylated reads. This analysis was conducted through a 5-fold cross-validation (Test 0-4) using 100,000 labeled examples of *E. coli* data.

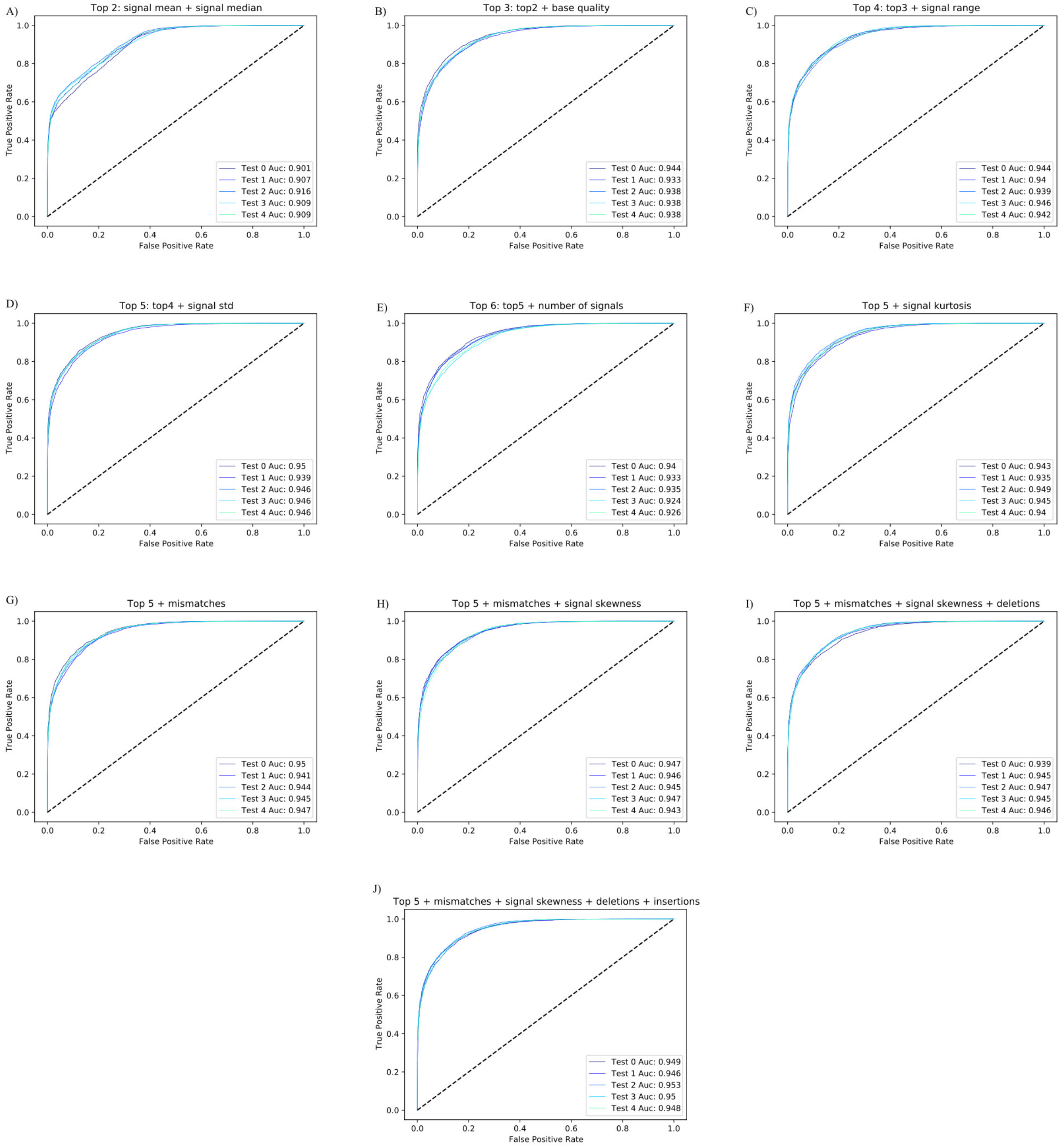

Figure S6: Incremental Feature Selection (IFS) combinations performance on a subset of the *E. coli* dataset. The rank of the 11 features by average AUC is 1. signal mean 2. signal median 3. base quality 4. signal range 5. signal std 6. number of signals 7. signal kurtosis 8. mismatches 9. signal skewness 10. deletions 11. insertions. The IFS strategy starts with **A)** signal mean and signal median and it adds one feature at a time to the combination for the model performance evaluation. **B, C, D, H, J)** The new feature is kept if it improves the performance. As an exception, Signal kurtosis is discarded due to its low AUC score shown in human data (S7). **E, G, I)** Otherwise, if the performance decreases, the new feature is discarded. **A-J)** Receiver operating characteristic (ROC) curve showing the false positive rate (x-axis) vs. the true positive rate (y-axis) for the read predictions on a mixture of methylated and unmethylated reads. This analysis was conducted through a 5-fold cross-validation (Test 0-4) using 100,000 labeled examples of *E. coli* data.

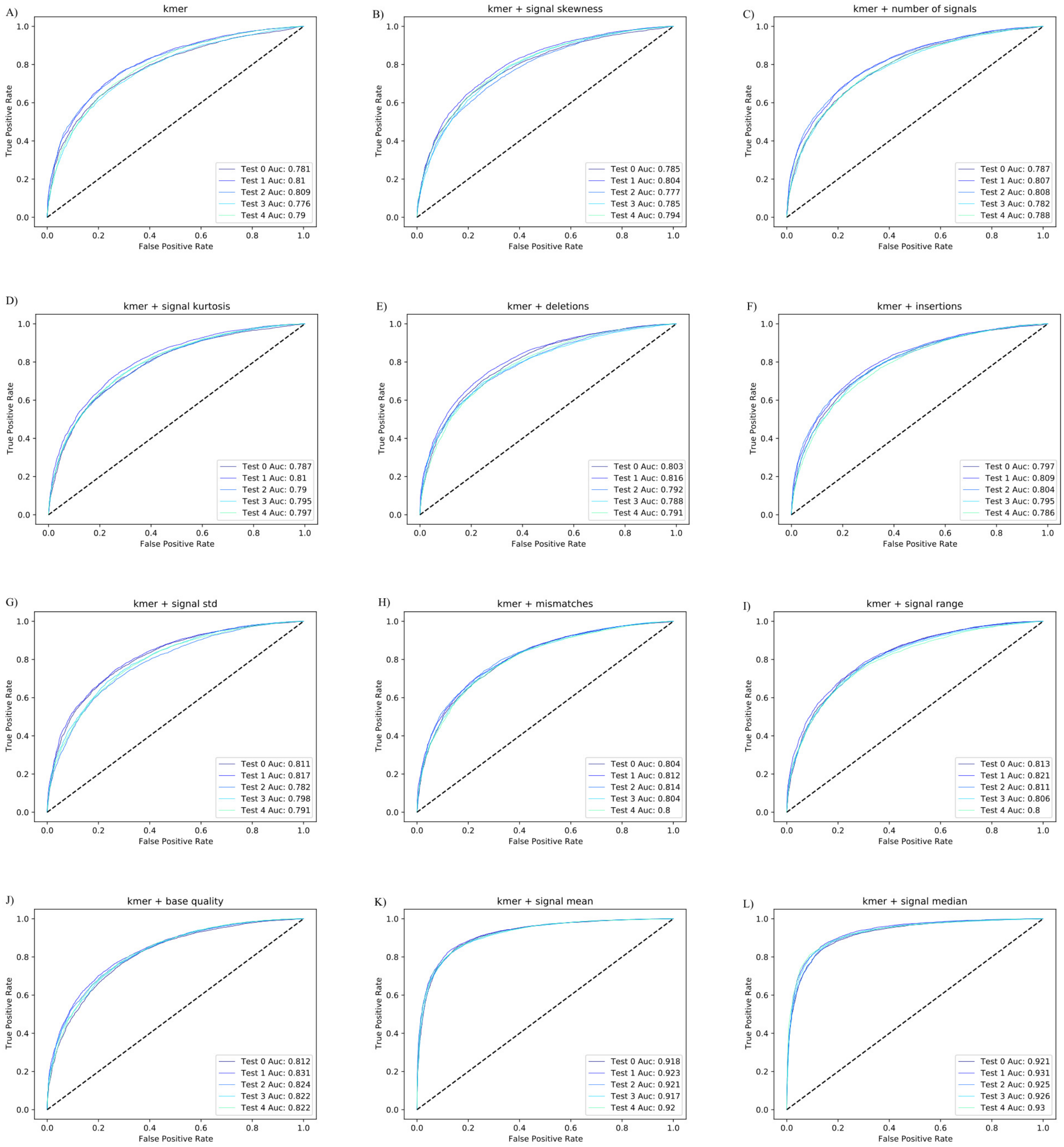

Figure S7: Single feature performance on a subset of the human dataset. Kmer serves as a baseline feature and it is combined with all other individual features. Performance on **A)** kmer alone, **B)** signal skewness, **C)** number of signals, **D)** signal kurtosis, **E)** deletions, **F)** insertions, **G)** signal std, **H)** mismatches, **I)** signal range, **J)** base quality, **K)** signal mean and **L)** signal median. **A-L)** Receiver operating characteristic (ROC) curve showing the false positive rate (x-axis) vs. the true positive rate (y-axis) for the read predictions on a mixture of methylated and unmethylated reads. This analysis was conducted through a 5-fold cross-validation (Test 0-4) using 100,000 labeled examples of human data.

Table S1: Nanopore sequencing datasets employed in the benchmarking and the number of reads corresponding to every dataset

Species	Name	Reads	Reference
E. coli	K12 ER2925 (Total)	181,137	Simpson et al. [13]
	M.SssI	69,899	
	Untreated	111,238	
H. sapiens	NA12878 (Norwich)	716,247	Jain et al. [2]
pUC19	(Total)	44,511	Rand et al. [12]
	Treated	17,219	
	Untreated	27,292	

Table S2: Cross validation per-read performance of DeepMP against Nanopolish, DeepSignal, Guppy, Megalodon and DeepMP Seq on *E. coli*.

Dataset	Test	Method	AUC	Accuracy	Precision	Recall	F-Score
E.coli	[0, 1000000]	Nanopolish	0.8783	0.7914	0.7476	0.8834	0.8099
		DeepSignal	0.9617	0.873	0.877	0.8644	0.8707
		Guppy	0.9236	0.8388	**0.9962**	0.654	0.7896
		Megalodon	0.9804	0.9354	0.99	0.8783	0.9308
		DeepMP Seq	0.9697	0.8687	0.8584	0.8762	0.8672
		DeepMP	**0.9869**	**0.9368**	0.9339	**0.9387**	**0.9363**
	[1000000, 2000000]	Nanopolish	0.8794	0.7966	0.7621	0.8844	0.8187
		DeepSignal	0.9711	0.8943	0.9049	0.8867	0.8958
		Guppy	0.9169	0.8242	**0.9961**	0.636	0.7763
		Megalodon	0.9789	0.9291	0.9895	0.8709	0.9264
		DeepMP Seq	0.9685	0.882	0.9217	0.8411	0.8795
		DeepMP	**0.9869**	**0.9372**	0.9351	**0.9418**	**0.9389**
	[2000000, 3000000]	Nanopolish	0.877	0.7886	0.7448	0.8873	0.8098
		DeepSignal	0.9749	0.903	0.9083	0.8989	0.9036
		Guppy	0.9213	0.83106	**0.9961**	0.6414	0.7803
		Megalodon	0.9801	0.933	0.9901	0.8762	0.9297
		DeepMP Seq	0.9709	0.8898	0.8971	0.8833	0.8902
		DeepMP	**0.9883**	**0.9387**	0.953	**0.9243**	**0.9385**
	[3000000, 4000000]	Nanopolish	0.8799	0.7939	0.7545	0.8879	0.8158
		DeepSignal	0.9754	0.9036	0.9158	0.8939	0.9048
		Guppy	0.9222	0.8303	**0.9966**	0.6458	0.7838
		Megalodon	0.980	0.9319	0.9906	0.8753	0.9294
		DeepMP Seq	0.9714	0.8911	0.8996	0.8863	0.8929
		DeepMP	**0.9884**	**0.9409**	0.9477	**0.9362**	**0.9419**
	[4000000, 5000000]	Nanopolish	0.8777	0.7943	0.7586	0.8867	0.8176
		DeepSignal	0.9737	0.9002	0.9116	0.8881	0.8997
		Guppy	0.9237	0.8366	**0.9957**	0.6557	0.7907
		Megalodon	0.9808	0.9343	0.9906	0.8781	0.9309
		DeepMP Seq	0.9699	0.8883	0.8914	0.8864	0.8889
		DeepMP	**0.9879**	**0.9397**	0.945	**0.9347**	**0.9398**

Table S3: Performance summary at read level of DeepMP against Nanopolish, DeepSignal, Guppy, Megalodon and DeepMP Seq on human and pUC19 datasets.

Dataset	Test	Method	AUC	Accuracy	Precision	Recall	F-Score
H. sapiens	Read level	Nanopolish	0.9284	0.908	0.9543	0.8949	0.9236
		DeepSignal	0.9629	0.9076	0.934	0.9191	0.9255
		Guppy	0.8602	0.768	0.9568	0.6565	0.7787
		Megalodon	0.9394	0.8788	**0.9594**	0.8418	0.8968
		DeepMP Seq	0.966	0.9146	0.9352	**0.9278**	0.9315
		DeepMP	**0.967**	**0.916**	0.9388	0.9260	**0.9324**
pUC19 plasmid	Read level	Nanopolish	0.7668	0.7211	0.6038	0.7737	0.6783
		DeepSignal	0.9123	0.8141	0.8207	0.7976	0.8090
		Megalodon	0.8901	0.7730	0.6583	**0.9547**	0.7792
		DeepMP Seq	0.935	0.8415	**0.857**	0.8108	0.8347
		DeepMP	**0.945**	**0.8697**	0.8370	0.9141	**0.8738**

References

[1] GCF52REF. *GpC methylation from fast5's to reference anchored calls. github.com/kpalin/gcf52ref. Retrieved June 2021.* 2020.

[2] Miten Jain et al. "Nanopore sequencing and assembly of a human genome with ultra-long reads". In: *Nature biotechnology* 36.4 (2018), pp. 338–345.

[3] Jared T Simpson. *Live methylation calling blog. simpsonlab.github.io/2020/03/03/nanopolish-v0.12.0/. Retrieved June 2021.* 2020.

[4] Jared T Simpson. *Quickstart - calling methylation with nanopolish nanopolish.readthedocs.io/en/latest/. Retrieved June 2021.* 2017.

[5] Huanle Liu et al. "Accurate detection of m 6 A RNA modifications in native RNA sequences". In: *Nature communications* 10.1 (2019), pp. 1–9.

[6] Qian Liu et al. "Detection of DNA base modifications by deep recurrent neural network on Oxford Nanopore sequencing data". In: *Nature communications* 10.1 (2019), pp. 1–11.

[7] Alexa BR McIntyre et al. "Single-molecule sequencing detection of N 6-methyladenine in microbial reference materials". In: *Nature communications* 10.1 (2019), pp. 1–11.

[8] Peng Ni et al. "DeepSignal: detecting DNA methylation state from Nanopore sequencing reads using deep-learning". In: *Bioinformatics* 35.22 (2019), pp. 4586–4595.

[9] ONT Guppy. *github.com/nanoporetech. Retrieved June 2021.* 2021.

[10] ONT Megalodon. *github.com/nanoporetech/megalodon. Retrieved June 2021.* 2021.

[11] ONT Rerio Models. *github.com/nanoporetech/rerio. Retrieved June 2021.* 2021.

[12] Arthur C Rand et al. "Mapping DNA methylation with high-throughput nanopore sequencing". In: *Nature methods* 14.4 (2017), pp. 411–413.

[13] Jared T Simpson et al. "Detecting DNA cytosine methylation using nanopore sequencing". In: *Nature methods* 14.4 (2017), pp. 407–410.

[14] Marcus Stoiber et al. "De novo identification of DNA modifications enabled by genome-guided nanopore signal processing". In: *BioRxiv* (2016), p. 094672.

[15] Zaka Wing-Sze Yuen et al. "Systematic benchmarking of tools for CpG methylation detection from Nanopore sequencing". In: *Nature Communications* 12.1 (2021), pp. 1–12.
